# LncRNA SNHG7/miR-34a-5p/SYVN1 axis plays a vital role in proliferation, apoptosis and autophagy in osteoarthritis

**DOI:** 10.1186/s40659-020-00275-6

**Published:** 2020-02-17

**Authors:** Feng Tian, Junhu Wang, Zhanhua Zhang, Jie Yang

**Affiliations:** 1 Department of Foot and Ankle Surgery, Honghui Hospital Affiliated to Xi’an Jiaotong University, No. 555 East Youyi Road, Xi’an, 710054 Shaanxi China; 2 Department of Internal Medicine, Honghui Hospital Affiliated to Xi’an Jiaotong University, Xi’an, Shaanxi China

**Keywords:** Osteoarthritis, SNHG7, miR-34a-5p, SYVN1, Cell growth, Autophagy

## Abstract

**Background:**

Osteoarthritis (OA) is one of the most common rheumatic diseases of which clinical symptoms includes swelling, synovitis and inflammatory pain, affect patients’ daily life. It was reported that non-coding RNAs play vital roles in OA. However, the regulation mechanism of ncRNA in OA pathogenesis has not been fully elucidated.

**Methods:**

The expression of SNHG7, miR-34a-5p and SYVN1 was detected using qRT-PCR in tissues, serum and cells. The protein expression of SYVN1, PCNA, cleavage-caspase 3, beclin1 and LC3 were measured using western blot. The RNA immunoprecipitation (RIP), RNA pulldown, and luciferase reporter assays were used to verify the relationship between SNHG7, miR-34a-5p and SYVN1. The MTT and flow cytometry assay was performed to detected cell proliferation and cell apoptosis respectively.

**Results:**

In this study, SNHG7 and SYVN1 expression were down-regulated, but miR-34a-5p was up-regulated in OA tissues and IL-1β treated cells compared with normal tissues and chondrocyte. Functional investigation revealed that up-regulated SNHG7 or down-regulated miR-34a-5p could promote cell proliferation and inhibit cell apoptosis and autophagy in OA cells. More than that, RIP, pulldown and luciferase reporter assay was applied to determine that miR-34a-5p was a target miRNA of SNHG7 and SYVN1 was a target mRNA of miR-34-5p. Rescue experiments showed that overexpression of miR-34a reversed high expression of SNHG7-mediated suppression of apoptosis and autophagy as well as promotion of proliferation, while its knockdown inhibited cell apoptosis and autophagy and promoted cell proliferation which could be impaired by silencing SYVN1. In addition, SNHG7 regulated SYVN1 through sponging miR-34a-5p.

**Conclusion:**

SNHG7 sponged miR-34a-5p to affect cell proliferation, apoptosis and autophagy through targeting SYVN1 which provides a novel sight into the pathogenesis of OA.

## Background

Osteoarthritis (OA) is one of the most common rheumatic diseases and one of the most common form of arthritis. Specific manifestations of osteoarthritis are joint swelling, synovitis and inflammatory pain, which affect patients’ work and daily life [1]. There are many causes of arthritis, including joint damage, joint or limb dysplasia, infection, obesity, age, and genetic factors [2, 3]. IL-1β is a member of the interleukin-1 cytokine family and is an important inflammatory cytokine involved in a variety of cellular processes, including proliferation, apoptosis [4, 5]. It has been previously reported that cellular inflammatory factors are widely involved in the pathogenesis of OA [6]. Moreover, IL-1β-induced OA cells resulted in the production of inflammatory factors, such as NO, PEG2 [4, 7]. At present, the pathogenesis of OA has not been fully elucidated.

Non-coding RNA (ncRNA) is composed of long non-coding RNA and small non-coding RNA. Among them, RNA with length over 200 nucleotides was defined as long non-coding RNA, and RNA with length less than 200 nucleotides was defined as small non-coding RNA [8]. Recently, studies have reported that ncRNAs have significant regulatory effects on human diseases [9]. Increasing evidence reported that ncRNA is involved in regulating the biological behavior of cells, including cell proliferation, invasion, metastasis, apoptosis and autophagy [10, 11]. Functional studies of lncRNA have shown that it can play an important role in cell development, differentiation and disease occurrence, including OA [12–14]. Notably, studies have reported that lncRNAs may acts as competitive RNA which competitively binds to target miRNAs to indirectly regulate mRNAs [15, 16]. lncRNA SNHG7 (small nucleolar RNA host gene 7) has been demonstrated to participate in cell development and progression of many diseases, especially in cancers [17–19]. Previous studies showed that SNHG7 was upregulated in several cancers, such as glioblastoma, colorectal cancer, gastric cancer and esophageal cancer [19, 20]. However, SNHG7 expression was down-regulated in OA [21], and the function and regulatory of SNHG7 in OA has not been fully explored.

MicroRNAs (miRNAs) are a class of small ncRNA that regulates post-transcriptional mRNA expression by binding 3′UTR of the target gene, leading to mRNA degradation or translation inhibition [22]. Multiple studies suggested that miRNAs play an indispensable role in cellular processes of many diseases, including cancers and inflammatory diseases [23–25]. However, the underlying regulatory network between ncRNA and mRNA in OA has not been fully elucidated.

miR-34a-5p has been reported to closely related to cell progression and signaling pathway in many disease [26–28]. In rat osteoarthritis model, inhibition of miR-34a-5p suppressed cell apoptosis [29]. However, the function of miR-34a-5p in OA has not been clearly understood.

In our study, we found that SNHG7 was down-regulated in OA tissues and IL-1β treated cells and investigated the function of SNHG7 in OA cell progression. In addition, we verify SNHG7 regulated SYVN1 expression by sponging miR-34a-5p in OA cellular process, providing novel insights into the pathogenesis of OA.

## Materials and methods

### Patients and specimens

The OA cartilage tissues were obtained from the knee joint of 15 OA patients undergoing total knee arthroplasty. The normal cartilage tissues were obtained from 10 patients undergoing femoral neck fracture without OA or rheumatic arthritis. Written informed consents were obtained from all patients and the study was approved by the Human Ethics Committee of Honghui hospital of Xi’an Jiaotong University.

### Cell culture and constructed OA cell model

To isolate primary human chondrocytes, cartilage specimens were cut into small pieces and dissolved by trypsin (Invitrogen, Carlsbad, CA, USA) collagenase Type II (Millipore Corp., Billerica, MA, USA) in Dulbecco’s modified Eagle’s medium (DMEM) for 10 h at 37 ℃ with 5% CO_2_. Isolated cells were obtained after filtration and cultured in DMEM/F12 medium contained with 10% fetal bovine serum (FBS) (Thermo, Waltham, MA, USA), 100 U/ml penicillin and 100 μg/ml streptomycin (Thermo), and incubated at 37 ℃ with 5% CO_2_. Third generation cells were obtained after subculture to construct the OA cell model.

Third generation cartilage cells were seeded in DMEM and treated with 10 ng/ml IL-1β (Sigma, St. Louis, MO, USA). Then incubated for 72 h at 37 ℃ with 5% CO_2_. The criterion for successful modeling was that the chondrocytes showed cytoplasmic retraction under the microscope and vacuoles were visible.

### Cell transfection

siSNHG7, siSYVN1 and their negative control (scramble) as well as pcDNA3.1-control (vector) and pcDNA3.1-SNHG7 (lncRNA SNHG7) were purchased from GenePharma (Shanghai, China). MiR-34a-5p inhibitor (anti-miR-34a-5p), miR-34a-5p mimics (miR-34a-5p) and their negative control (anti-NC and NC) were also purchased from GenePharma (Shanghai, China). These plasmids and oligos were transfected into OA cells using Lipofectamine 3000 Reagents (Invitrogen).

### Quantitative real-time PCR (qRT-PCR) assay

Total RNA was extracted from tissues, serums and cells with TRIzol reagent (Invitrogen) according to the manufacturer’s protocols. For the miRNA, total RNA (1 μg) were reverse transcribed into cDNA with TaqMan microRNA assay (Applied Biosystems, Carlsbad, CA, USA) according to manufacturer’s instructions. For the mRNA, total RNA (1 μg) was reverse transcribed into cDNA using M-MLV Reverse Transcriptase (Invitrogen). qRT-PCR was performed using SYBR^®^ Green (Promega, Madison, WI, USA). U6 and GAPDH were used to as reference gene. The primers used for qRT-PCR are: SNHG7 forward: 5′-GTGACTTCGCCTGTGATGGA-3′, reverse: 5′-GGC CTCTATCTGTACCTTTATTCC-3′; SYVN1 forward: 5′-CTTCGTCAGCCACGCTTATC-3′ reverse: 5′-CCACGGAGTGCAGCACATAC-3′; GAPDH forward: 5′-AACGTGTCAGTGGTGGACCTG-3′, reverse: 5′-AGTGGGTGTCGCTGTTGAAGT-3′; miR-34a-5p: Forward: 5′-ACACTCCAGCTGGGTGGCAGTGTCTTAGC, reverse: 5′-CTCAACTGGTGTCGTGGAGTCGGCAATTCAGTTGAGACAACCA-3′; U6 forward: 5′-CTCGCTTCGGCAGCACA-3′, reverse: 5′-AACGCTTCACGAATTTGCGT-3′.

Fluorescence was detected in an ABI 7300 System (Applied Biosystems). The 2^−ΔΔCt^ method was used to analyze the relative expression of mRNA, miRNA, and lncRNA.

### Western blot

Total protein was separated from cells by SDS-PAGE and then was transferred to a polyvinylidene difluoride membrane (Millipore, Bedford, MA, USA). Then membranes were blocked in TBS with 5% no-fat milk and incubated with primary antibodies, SYVN1, PCNA, cleaved-caspase 3, beclin1, LC3 and GAPDH (1:2000, Santa Cruz Biotechnology Inc., Santa Cruz, CA, USA). The membranes were incubated with HRP-conjugated secondary antibodies (1:1000, Santa Cruz Biotech, Santa Cruz, CA). The blots were detected using Applied the PierceTM ECL western blotting substrate (ThermoFisher Scientific, Inc., Waltham, MA, USA) according to the manufacturer’s protocols.

### Cell proliferation and cell apoptosis

Cell proliferation was measured using 3-(4, 5-dimethyl-2-thiazolyl)-2, 5-diphenyl-2Htetrazolium bromide (MTT) assay. 2 × 10^3^ cells were seeded in 96-well cell culture plates (Corning Inc., Corning, NY, USA) per well. Then each well was added into 20 μL MTT (5 g/L, Sigma) and incubated for 4 h at 37 ℃. Subsequently, 150 μL dimethyl sulfoxide (DMSO, Sigma) was added into each well. Wrapped the plate in foil and shook for 15 min. A spectrophotometric microplate reader (Beyotime Institute of Biotechnology, Haimen, China) was applied to detect cell proliferation at a wavelength of 450 nm.

Cell apoptosis was measured using flow cytometry. Cells were collected and washed with PBS. Then cells were stained with the PI/FITC-Annexin V. After incubated for 30 min at 37 ℃ without light, cell apoptosis was detected by flow cytometry (BD FACS Aria; BD Biosciences, Franklin Lakes, NJ, USA).

### Enzyme-linked-immunosorbent-assay (ELISA)

The IL-1β level was measured by ELISA using IL-1β ELISA kit (R&D, Shenzhen Jingmei Lrd. China) according to manufacturer’s instructions. Control buffer and serums samples were added into wells of ELISA plates contained antibody IL-1β and then were incubated for 2 h at room temperature. After washed, 100 μL the secondary antibody conjugated HRP was added into each well and incubated for 2 h. Then washed again, 100 μL treated TMB was added into each well and incubated for 15 min without light. The reaction was stopped with lN H_2_SO_4_ and the absorbance was measured at 450 nm.

### RNA immunoprecipitation (RIP) and RNA pulldown assay

The RIP and RNA pulldown assays were used to verify the binding relationship between SNHG7 and miR-34a-5p or miR-34a-5p and SYVN1. The RIP assay was performed using the Magna RIP™ RNA Binding Protein Immunoprecipitation Kit (Millipore, USA) according to the manufacturer’s instructions. Cells were lysed in RIP lysis buffer, and then were incubated with RIP buffer with a human anti-Ago2 antibody (Abcam, Cambridge, MA, USA). Each sample was incubated with proteinase K to digest protein. Purify RNA was obtained and then was analyzed using qRT-PCR assay. For the RNA pulldown assay, 3′biotinylated miR-34a-5p or 3′biotinylated miR-NC was transfected into OA cells. Cells were harvested after transfection 48 h and then were lysed and incubated in the lysis buffer. The complexes were isolated by streptavidin agarose beads. Then the expressions of SNHG7 in pulldown samples were measured by qRT-PCR assay.

### Luciferase reporter assay

3′UTR of SNHG7 or 3′UTR of SYVN1 was amplified by PCR and inserted into pMIR-REPORT™ (ThermoFisher Scientific, USA) to construct SNHG7 wild type (SNHG7-wt) or SYVN1 wild type (SYVN1-wt) respectively. The mutant type (SNHG7-mut) or (SYVN1-mut) was made with GeneArt™ Site-Directed Mutagenesis PLUS System (ThermoFisher Scientific, USA). Then SNHG7-wt or SNHG7-mut was co-transfected with NC or miR-34a-5p into HEK-293 K cells. SNHG7-wt or SNHG7-mut was co-transfected with anti-NC or anti-34a-5p into HEK-293 K cells. In addition, SYVN1-wt or SYVN1-mut was co-transfected with NC or miR-34a-5p into HEK-293 K cells. At 48 h after transfection, the luciferase activity was measured using the using the dual-luciferase reporter assay system (Promega) following the protocols.

### Statistical analysis

The data were presented as mean ± SD expect where indicated otherwise. Student t-test was used to assess all comparisons groups. Pearson’s correlation analysis was applied to analyze statistical correlation. A *P* value < 0.05 was considered to be statistically significant. The results were displayed using GraphPad Prism 7.0 (GraphPad Software, San Diego, CA, USA).

## Results

### SNHG7 was down-regulated in OA tissues

**Fig. 1:**
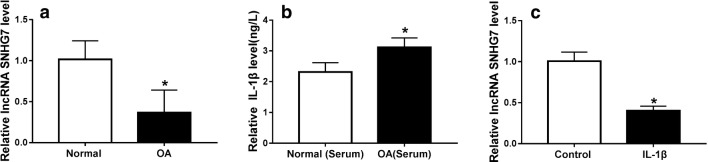
SNHG7 expressed less in OA tissues. **a** The expression of SNHG7 in OA tissues and normal tissues was detected by qRT-PCR. **b** The IL-1β level in OA serum and normal serum were measured by ELISA assay. **c** The expression of SNHG7 in OA cells stimulated with 10 ng/ml IL-1β and OA cells. *P < 0.05

To investigate the role of SNHG7 in OA, qRT-PCR was used to detect the expression of SNHG7 in OA tissues obtained from 15 OA patients and normal tissues obtained from 10 trauma patients. The results showed that SNHG7 was down-regulated in OA tissues compared with that in normal tissues (Fig. 1a). Then, the IL-1β level was measured using ELISA assay. We found that the IL-1β level of OA group was significantly higher than that of normal group (Fig. 1b). To construct OA model in vitro, articular chondrocytes (ACs) were extracted from knee joints of OA patients and stimulated with 10 ng/ml IL-1β to simulate ACs. We found that the expression of SNHG7 was significantly decreased in ACs after IL-1β treatment (Fig. 1c). Normal chondrocytes were isolated from patients undergoing femoral neck fracture without OA or rheumatic arthritis. We detected the SNHG7 expression in normal chondrocytes and normal chondrocytes treated with IL-1β and found that SNHG7 was downregulated in IL-1β-treated normal chondrocytes, but the percentage of downregulation was much smaller than that in IL-1β-treated OA cells (Additional file 1: Figure S1). Therefore, the results revealed that SNHG7 was associated with OA.

### Overexpression of SNHG7 promoted cell proliferation and inhibited cell apoptosis and autophagy

**Fig. 2:**
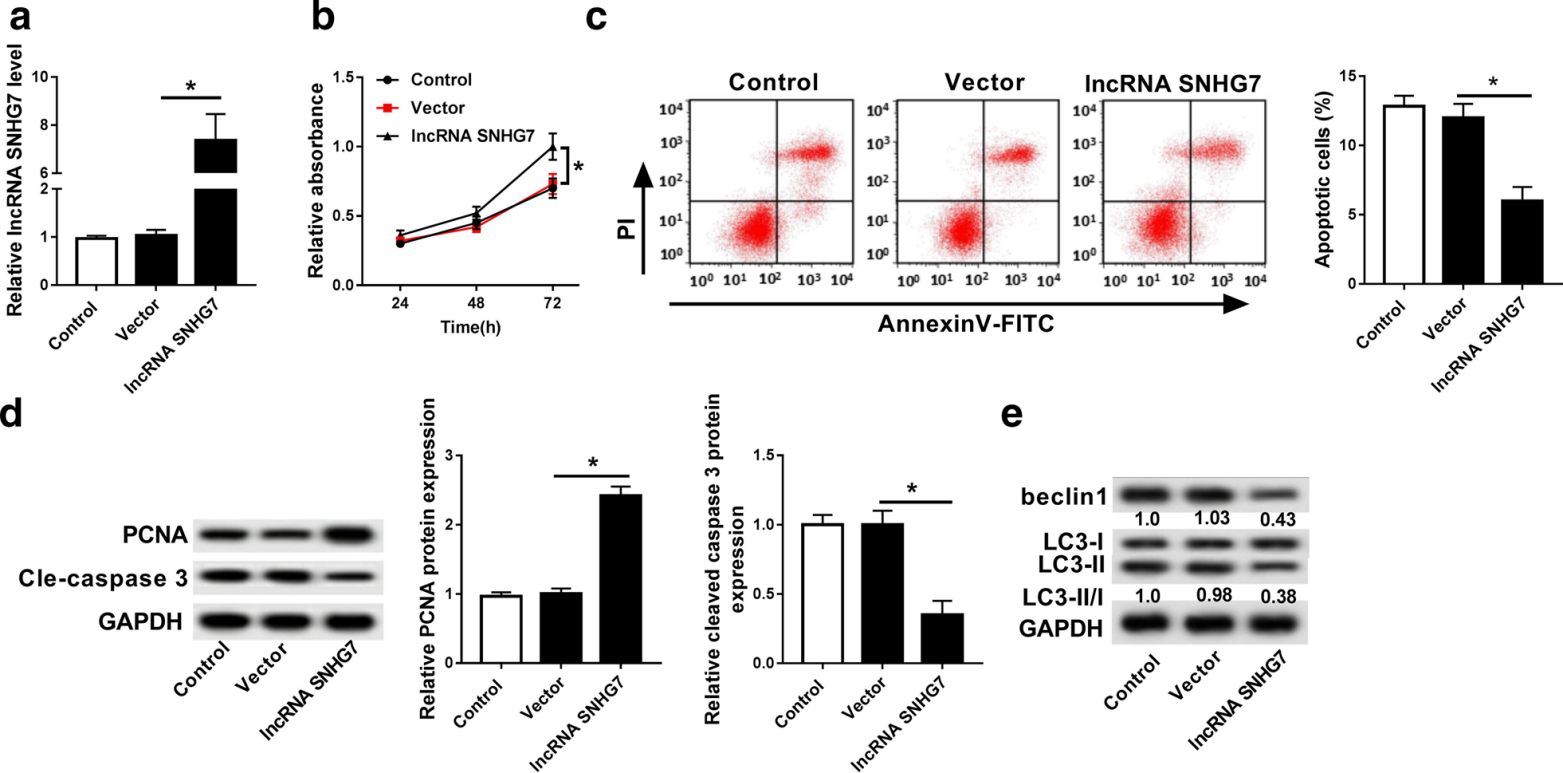
Overexpression of SNHG7 promoted cell proliferation as well as inhibited cell apoptosis and autophagy. **a** The expression of SNHG7 was detected in OA cells transfected with control, vector and lncRNA SNHG7 by qRT-PCR. **b** Cell proliferation was measured in OA cells (IL-1β) transfected with control, vector and lncRNA SNHG7 after transfection 24 h, 48 h, 72 h by MTT assay. **c** Cell apoptosis was detected in OA cells (IL-1β) transfected with control, vector and lncRNA SNHG7 by flow cytometry. **d** The protein expression of PCNA and cleaved-caspase 3 were measured in OA cells (IL-1β) transfected with control, vector and lncRNA SNHG7 by western blot. **e** The protein expression of beclin1 and LC3 were measured in OA cells (IL-1β) transfected with control, vector and lncRNA SNHG7 by western blot. *P < 0.05

As shown in Additional file 1: Figure S2A, we observed the successful overexpression efficiency of lnc RNA SNHG7 in normal chondrocytes. Moreover, overexpression of lnc RNA SNHG7 dramatically promoted cell proliferation and inhibited cell apoptosis in normal chondrocytes treated with IL-1β (Additional file 1: Figure S2B, C). To examine the function of SNHG7 in OA, we overexpressed SNHG7 in OA cells (Fig. 2a). Then MTT assay demonstrated that overexpression of SNHG7 significantly promoted cell proliferation (Fig. 2b). The flow cytometry assay showed that the apoptotic cells marked as Annexin V positive in lncRNA SNHG7 group were obviously less than that in control and vector groups (Fig. 2c). Moreover, SNHG7 expression increased the protein expression of PCNA, whereas decreased cleavage caspase-3 (Fig. 2d). Furthermore, lncRNA SNHG7 transfection remarkably reduced the protein expression of beclin1 and LC3, indicating SNHG7 overexpression inhibited cell autophagy (Fig. 2e). These findings showed that overexpression of SNHG7 could promote cell proliferation and inhibit cell apoptosis and autophagy in OA.

### miR-34a-5p inhibitor promoted cell proliferation as well as inhibited cell apoptosis and autophagy

**Fig. 3:**
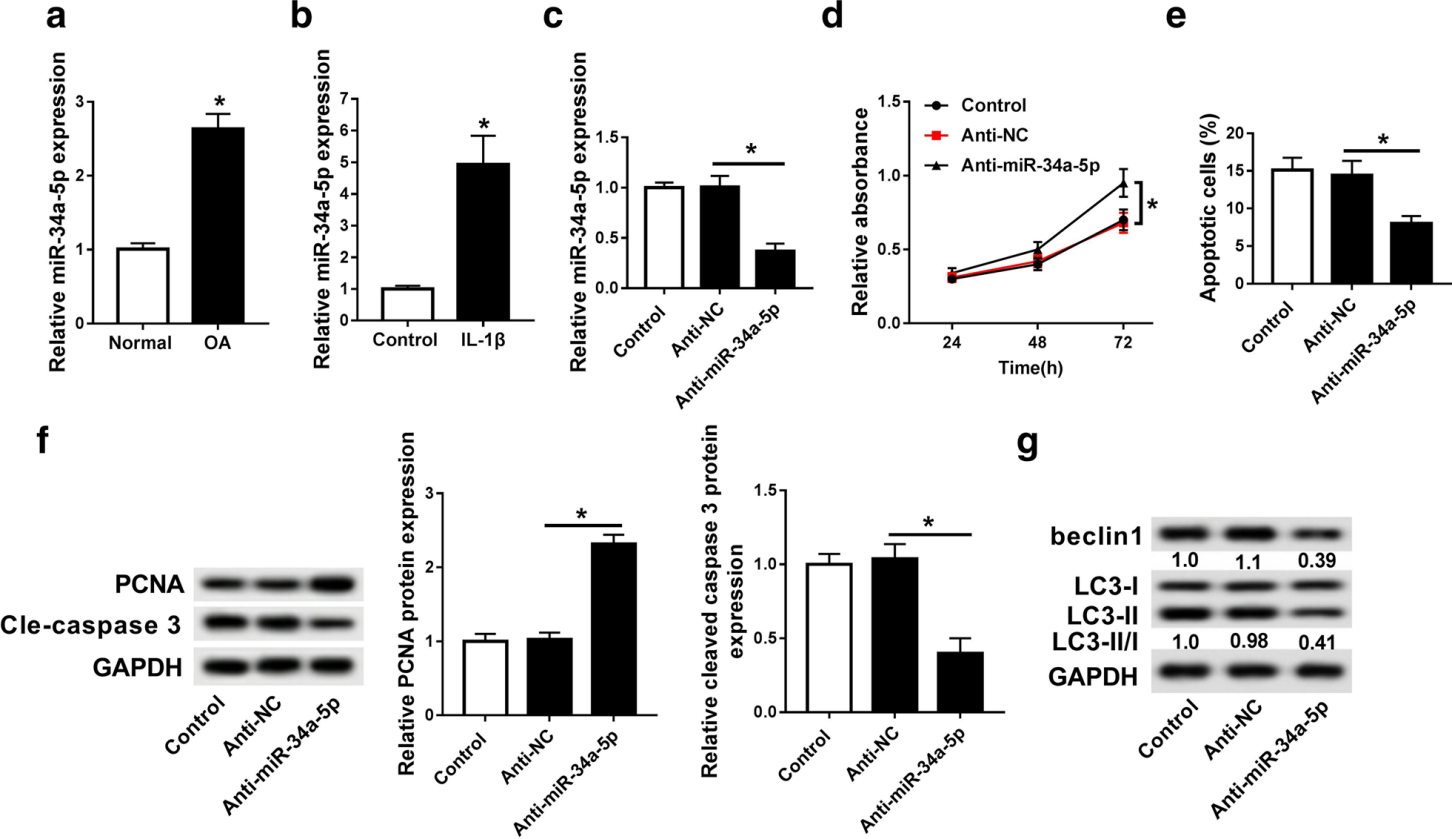
Down-regulation of miR-34a-5p promoted cell proliferation as well as inhibited cell apoptosis and autophagy. **a** The expression of miR-34a-5p in OA tissues and normal tissues was detected by qRT-PCR. **b** The expression of miR-34a-5p in OA cells stimulated 10 ng/ml IL-1β and OA cells. **c** The expression of miR-34a-5p was detected in OA cells transfected with control, anti-NC and anti-miR-34a-5p by qRT-PCR. **d** Cell proliferation was measured in OA cells (IL-1β) transfected with control, anti-NC and anti-miR-34a-5p after transfection 24 h, 48 h, 72 h by MTT assay. **e** Cell apoptosis was detected in OA cells (IL-1β) transfected with control, anti-NC and anti-miR-34a-5p by flow cytometry. **f** The protein expression of PCNA and cleaved-caspase 3 were measured in OA cells (IL-1β) transfected with control, anti-NC and anti-miR-34a-5p by western blot. **g** The protein expression of beclin1 and LC3 were measured in OA cells (IL-1β) transfected with control, anti-NC and anti-miR-34a-5p by western blot. *P < 0.05

Previous study reported that miR-34a was a target miRNA of SNHG7 in colorectal cancer. In our study, we found that miR-34a-5p was up-regulated in OA tissues compared with that in normal tissues (Fig. 3a). In addition, the expression of miR-34a-5p was significantly increased in ACs stimulated by IL-1β (Fig. 3b). Thus, anti-miR-34a-5p was transfected into OA cells to investigate the function of miR-34a-5p in OA. As shown in Fig. 3c, we observed that miR-34a-5p expression was much less in anti-miR-34a-5p group compared with that in control and anti-NC groups. Furthermore, MTT assay showed that anti-miR-34a-5p obviously promoted cell proliferation (Fig. 3d). The analysis of flow cytometry indicated that cell apoptosis was inhibited by down-regulation of miR-34a-5p (Fig. 3e). In addition, PCNA protein expression was significantly induced and cleavage-caspase 3 was dramatically decreased by anti-miR-34a-5p (Fig. 3f). More than that, miR-34a-5p knockdown obviously decreased beclin 1 protein expression accompanied with decreased LC3-II/LC3-I ratio (Fig. 3g). Therefore, these results confirmed that down-regulated miR-34a-5p expression could promote cell proliferation and impede cell apoptosis and autophagy.

### MiR-34-5p was a target miRNA of SNHG7

**Fig. 4:**
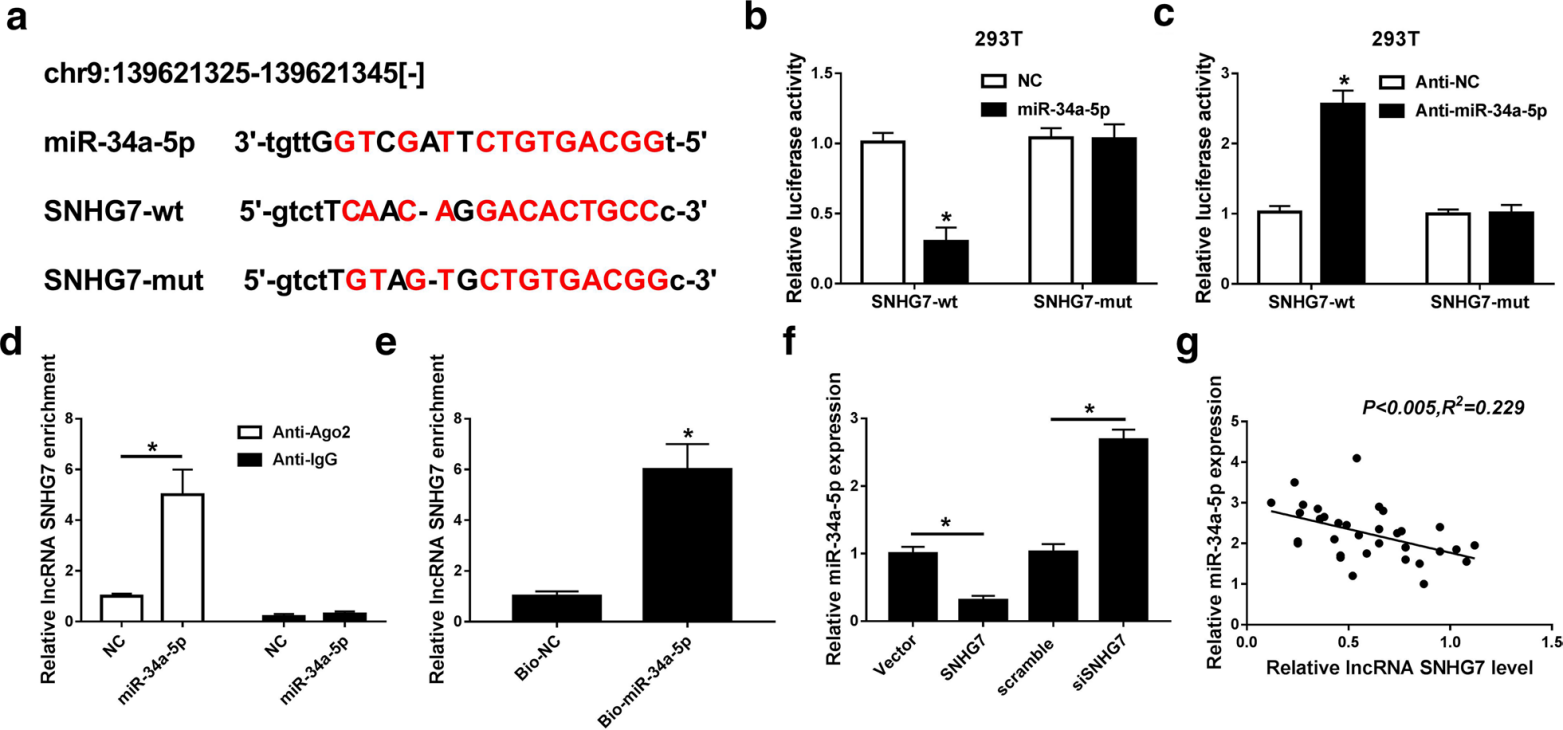
miR-34-5p was a target miRNA of SNHG7. **a** The predicted binding sites of miR-34a-5p to SNHG7 sequence using starBase 2.0. **b** The luciferase activity of 293T cells co-transfected with miR-34a-5p and luciferase reporters containing SNHG7-wt or SNHG7-mut was detected using luciferase reporter assay. **c** The luciferase activity of 293T cells co-transfected with anti-miR-34a-5p and luciferase reporters containing SNHG7-wt or SNHG7-mut was detected using luciferase reporter assay. **d** The expression of SNHG7 was detected in the samples bound to the Ago2 antibody by RIP assay. **e** The expression of SNHG7 was detected using qRT-PCR in samples pulled down by biotinylated miR-34a-5p or negative control (NC). **f** The expression of miR-34a-5p was detected in OA cells (IL-1β) transfected with control, vector, SNHG7, scramble and siSNHG7 by qRT-PCR. **g** Pearson’s correlation analysis revealed the significant negative correlation between SNHG7 and miR-34a-5p expression. *P < 0.05

To verify the relationship between miR-34-5p and SNHG7, online database starBase 2.0 was used to predict our hypothesis. We found that miR-34a-5p was a potential miRNA target of SNHG7 (Fig. 4a). Then luciferase reporter assay displayed that when the miR-34a-5p bound to the SNHG7-wt, the luciferase activity was significantly reduced, but not with SNHG7-mut. Inversely, when the anti-miR-34a-5p bound to SNHG7-wt rather than SNHG7-mut, the luciferase activity was significantly increased (Fig. 4b, c). Furthermore, the RIP and pull-down assays further verified that SNHG7 directly bound to miR-34a-5p because a significant amount of SNHG7 was measured (Fig. 4d, e). In addition, SNHG7 transfection markedly reduced miR-34a-5p expression while siSNHG7 obviously promoted miR-34a-5p expression (Fig. 4f). More than that, Pearson’s correlation analysis suggested SNHG7 expression was significant negative correlated with miR-34a-5p expression in OA tissues (Fig. 4g). Taken together, SNHG7 directly targeted miR-34a-5p.

### Overexpression of miR-34a-5p reversed the effect of SNHG7 in OA cells

**Fig. 5:**
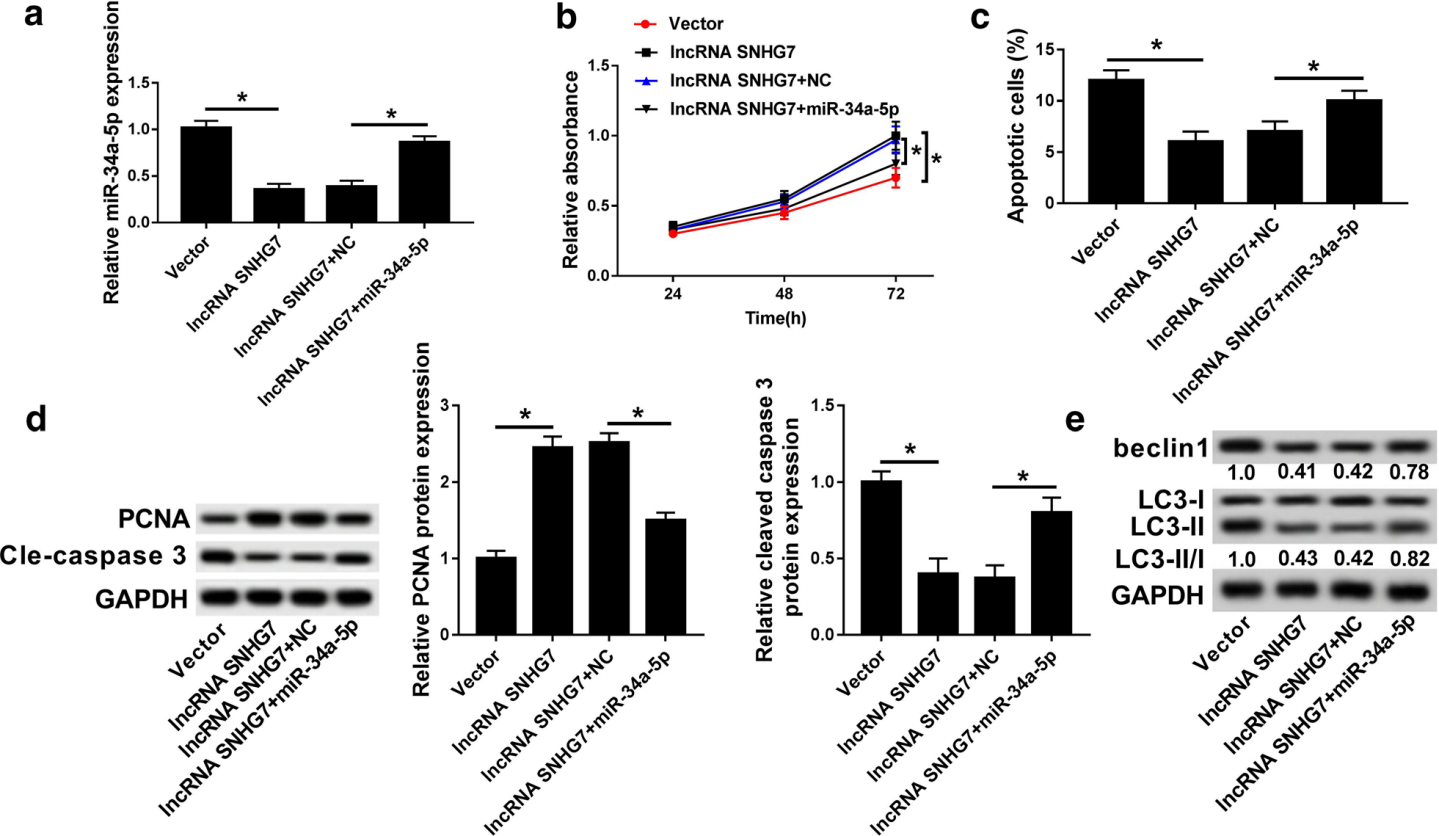
Overexpression of miR-34a-5p reversed the effect of SNHG7 on OA cells. **a** The expression of miR-34a-5p was detected in OA cells transfected with vector, lncRNA SNHG7, lncRNA SNHG7 + NC and lncRNA SNHG7 + miR-34a-5p by qRT-PCR. **b** Cell proliferation was measured in OA cells (IL-1β) transfected with vector, lncRNA SNHG7, lncRNA SNHG7 + NC and lncRNA SNHG7 + miR-34a-5p after transfection 24 h, 48 h, 72 h by MTT assay. **c** Cell apoptosis was detected in OA cells (IL-1β) transfected with vector, lncRNA SNHG7, lncRNA SNHG7 + NC and lncRNA SNHG7 + miR-34a-5p by flow cytometry. **d** The protein expression of PCNA and cleaved-caspase 3 were measured in OA cells (IL-1β) transfected with vector, lncRNA SNHG7, lncRNA SNHG7 + NC and lncRNA SNHG7 + miR-34a-5p by western blot. **e** The protein expression of beclin1 and LC3 were measured in OA cells (IL-1β) transfected with vector, lncRNA SNHG7, lncRNA SNHG7 + NC and lncRNA SNHG7 + miR-34a-5p by western blot. *P < 0.05

To further investigate the regulatory network of SNHG7 and miR-34a-5p, the miR-34a-5p and lncRNA SNHG7 was co-transfected into OA cells. Interestingly, as shown in Fig. 5a, the expression of miR-34a-5p was significantly decreased in lncRNA SNHG7 group compared with that in vector group, whereas co-transfection of lncRNA SNHG7 and miR-34a-5p increased miR-34-5p expression. Additionally, overexpression of SNHG7 promoted cell proliferation and inhibited cell apoptosis, which was impaired by up-regulation of miR-34a-5p (Fig. 5b, c). More than that, miR-34a-5p overexpression reversed the effects of overexpression of SNHG7 on PCNA, cleaved- caspase 3, beclin1 and LC3 in OA (Fig. 5d, e). These findings revealed that up-regulated miR-34a-5p reversed the effects of SNHG7 on OA cell growth, apoptosis and autophagy.

### SYVN1 was a target mRNA of miR-34a-5p

**Fig. 6:**
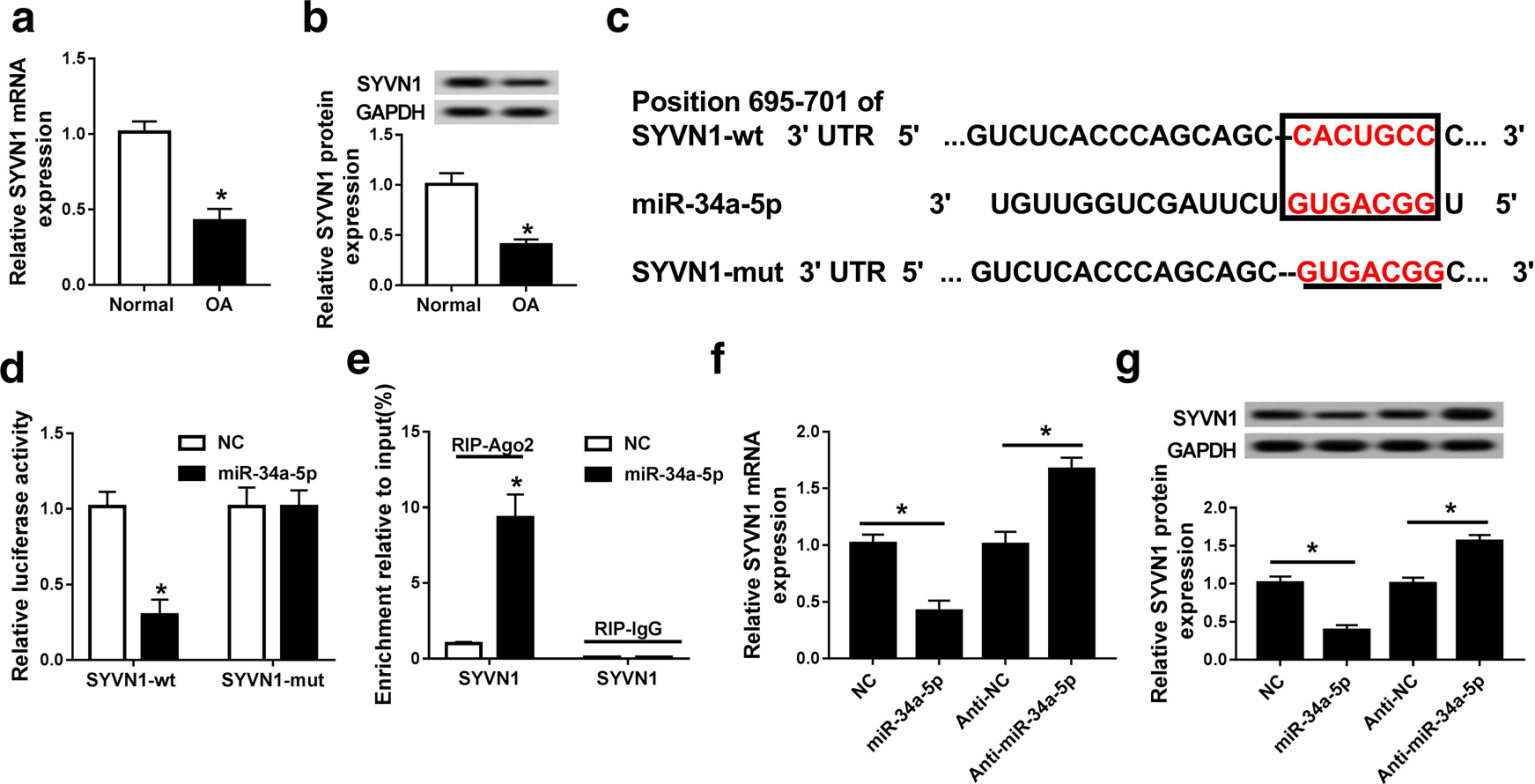
SYVN1 was a target mRNA of miR-34a-5p. **a** The expression of SYVN1 in OA tissues and normal tissues was detected by qRT-PCR. **b** The expression of SYVN1 in OA cells stimulated 10 ng/ml IL-1β and OA cells. **c** The predicted binding sites of SYVN1 to miR-34a-5p sequence using starBase 2.0. **d** The luciferase activity of 293T cells cotransfected with miR-34a-5p and luciferase reporters containing SYVN1-wt or SYVN1-mut was detected using luciferase reporter assay. **e** The expression of SYVN1 was detected in the samples bound to the Ago2 antibody or IgG by RIP assay. **f**, **g** The mRNA **f** and protein **g** expression of SYVN1 was detected in OA cells (IL-1β) transfected with NC, miR-34a-5p, anti-NC and anti-miR-34a-5p by qRT-PCR and western blot. *P < 0.05

As shown in Fig. 6a, b, SYVN1 expression was significantly decreased in OA tissues. Notably, SYVN1 was firstly identified as a potential target of miR-34a-5p using bioinformatics analysis (Fig. 6c). The luciferase report assay was applied to verify miR-34a-5p directly targeted SYVN1 in 293T cells. The results showed the luciferase activity was obviously reduced when SYVN1-wt and miR-34a-5p mimic were transfected into 293T cells, while SYVN1-mut had no effect (Fig. 6d). RIP assay showed that the endogenous SYVN1 was significantly enrichment in OA cells co-transfected miR-34a-5p and SYVN1 (Fig. 6e), revealing the direct binding between SYVN1 and miR-34a-5p. Besides, miR-34a-5p transfection inhibited the mRNA and protein expression of SYVN1, whereas anti-34a-5p transfection induced SYVN1 mRNA and protein expression in OA cells (Fig. 6f, g). Therefore, the results suggested that SYVN1 was a target of miR-34a-5p.

### SYVN1 knockdown impaired the effect on cell proliferation, apoptosis and autophagy caused by anti-miR-34a-5p

**Fig. 7:**
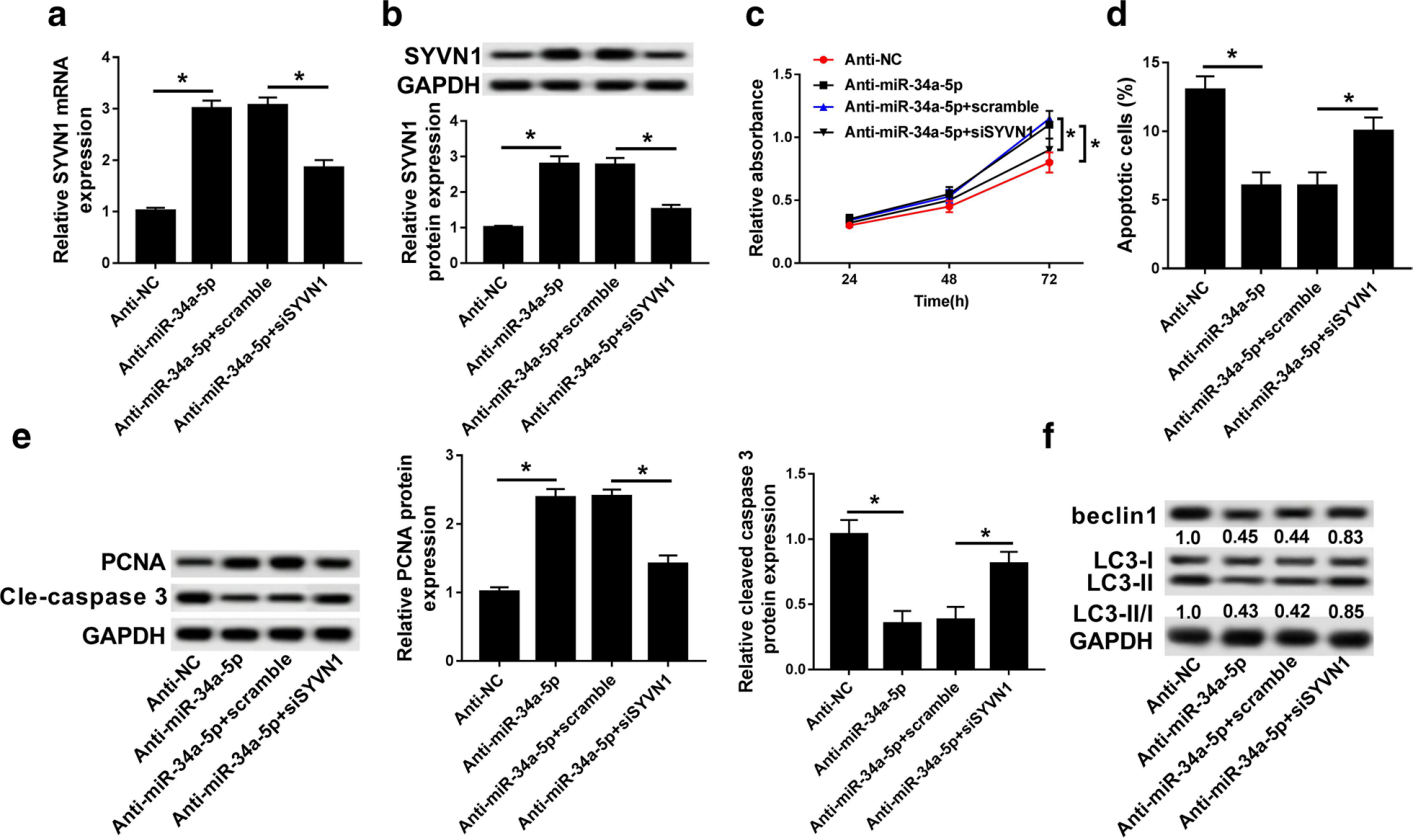
SYVN1 knockdown impaired the effect on cell proliferation, apoptosis and autophagy caused by anti-miR-34a-5p. **a**, **b** The mRNA **a** and protein **b** expression of SYVN1 was detected in OA cells (IL-1β) transfected with anti-NC, anti-miR-34a-5p, anti-miR-34a-5p + scramble and anti-miR-34a-5p + siSYVN1 by qRT-PCR and western blot. **c** Cell proliferation was measured in OA cells (IL-1β) transfected with anti-NC, anti-miR-34a-5p, anti-miR-34a-5p + scramble and anti-miR-34a-5p + siSYVN1 after transfection 24 h, 48 h, 72 h by MTT assay. **d** Cell apoptosis was detected in OA cells (IL-1β) transfected with anti-NC, anti-miR-34a-5p, anti-miR-34a-5p + scramble and anti-miR-34a-5p + siSYVN1 by flow cytometry. **e** The protein expression of PCNA and cleaved-caspase 3 were measured in OA cells (IL-1β) transfected with anti-NC, anti-miR-34a-5p, anti-miR-34a-5p + scramble and anti-miR-34a-5p + siSYVN1 by western blot. **f** The protein expression of beclin1 and LC3 were measured in OA cells (IL-1β) transfected with anti-NC, anti-miR-34a-5p, anti-miR-34a-5p + scramble and anti-miR-34a-5p + siSYVN1 by western blot. *P < 0.05

To determine whether miR-34a-5p regulated cell processes through SYVN1, we conducted rescue experiments to verify our hypothesis. In the present study, the mRNA and protein expression of SYVN1 was induced by anti-miR-34a-5p transfection, which inhibited by knockdown of SYVN1 (Fig. 7a, b). Besides, down-regulated miR-34a-5p not only promoted cell proliferation and inhibited cell apoptosis, but also reduced cleavage-caspase 3, beclin1 and LC3 proteins expression and induced PCNA protein expression. However, these effects were abolished by siSYVN1 (Fig. 7c–f). These findings suggested that low expressed miR-34a-5p promoted cell progression and inhibited cell apoptosis and autophagy, which was rescued by knockdown of SYVN 1.

### SNHG7 sponged miR-34a-5p to regulate SYVN1 expression in OA

**Fig. 8:**
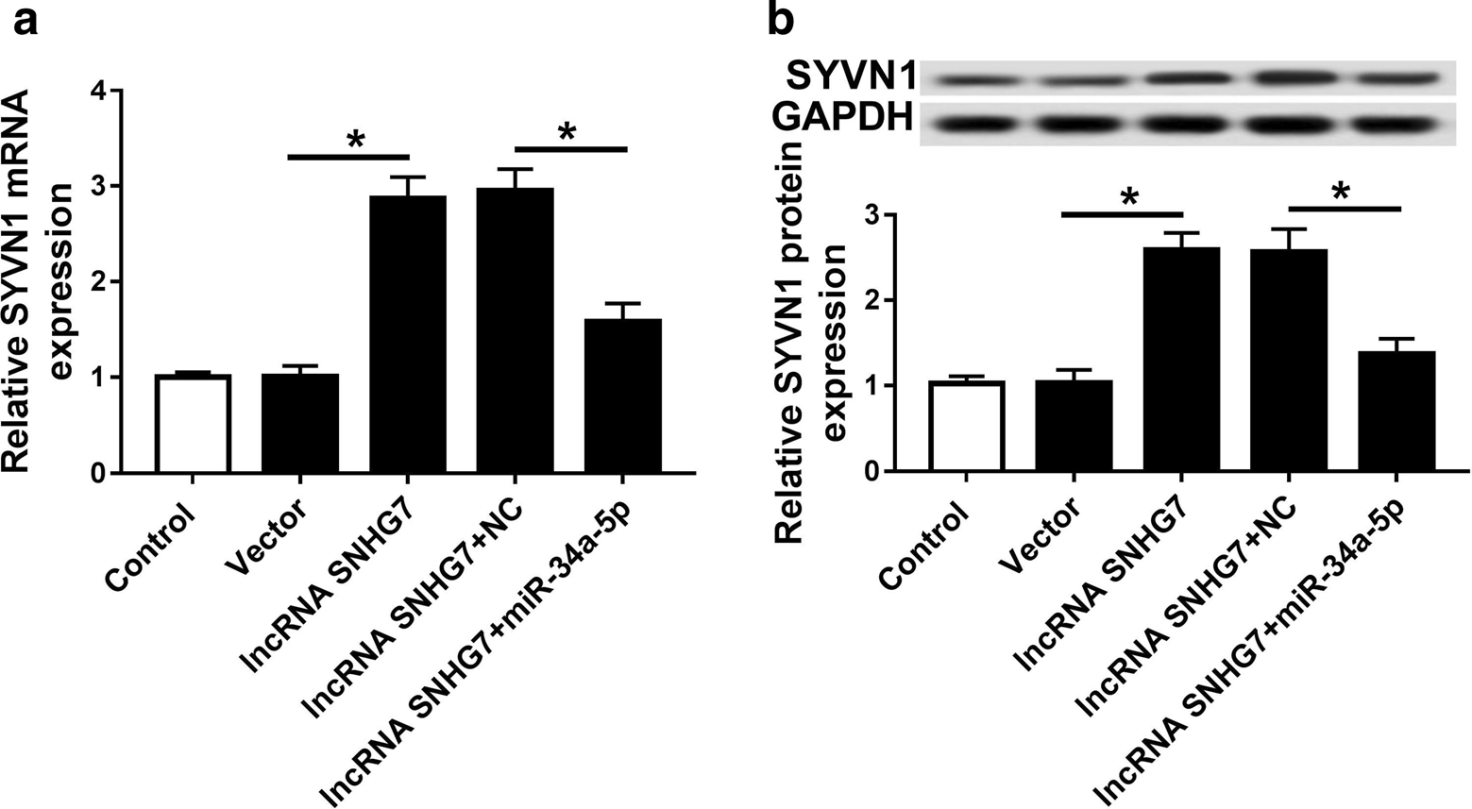
SNHG7 sponged miR-34a-5p to regulate SYVN1 expression in OA cells. **a**, **b** The mRNA **a** and protein **b** expression of SYVN1 was detected in OA cells (IL-1β) transfected with control, vector, lncRNA SNHG7 + NC and lncRNA SNHG7 + miR-34a-5p by qRT-PCR and western blot. *P < 0.05

To validate the regulatory network of SNHG7, miR-34a-5p and SYVN1, we detected SYVN1 expression in OA cells transfection with control, vector, lncRNA SNHG7, lncRNA SNHG7 + NC and lncRNA SNHG7 + miR-34a-5p. As shown in Fig. 8a, b, lncRNA SNHG7 transfection obviously promoted SYVN1 mRNA and protein expression in comparison with control and vector transfections. Interestingly, the mRNA and protein expression of SYVN1 in lncRNA SNHG7 + miR-34a-5p group was remarkably decreased compared with that in lncRNA SNHG7 + NC group. Thus, miR-34a-5p expression could decrease SYVN1 mRNA and protein expression induced by SNHG7 overexpression.

## Discussion

Multiple studies have suggested that lncRNAs are associated with cell progression in OA and the function and regulatory mechanism of several lncRNAs was investigated in OA [15, 30]. For example, SNHG5 promoted chondrocyte cell proliferation and migration and functioned as a ceRNA to regulate SOX2 expression through sponging miR-26a in OA [15]. In addition, down-regulated MEG3 could affect OA progression through miR-16/SMAD7 axis [31]. lncRNA SNHG7 was reported to as an oncogene in several cancers [17, 18, 20]. However, contrary to the previous study [21], we found that SNHG7 expression was reduced in OA tissues compared with normal tissues and the level of IL-1β was increased in OA serum compared with normal serum. Then we constructed the OA cell model. In this model, SNHG7 expressed lower in OA cells treated with IL-1β compared with control. Next, the results of MTT, flow cytometry and western blot showed that overexpression of SNHG7 promoted OA cell proliferation and inhibited cell apoptosis and autophagy, implying that SNHG7 was involved in OA progression and autophagy.

Emerging evidence reported that miR-34a-5p, as a target of lncRNA, plays an important role in cell progression in diseases, such as nasopharyngeal carcinoma, osteosarcoma and OA. For example, XIST was involved in cancer metastasis and development through targeting miR-34a-5p in human nasopharyngeal carcinoma [32]. C2dat1 contributed to cell proliferation, migration and invasion through targeting miR-34a-5p in osteosarcoma cells [33]. In OA, lncRNA UFC1 contributed to chondrocyte proliferation by association with miR-34a [34]. Additionally, miR-34a expression was induced by IL-1β in OA [31]. Consistent with previous studies [35], we found that miR-34a-5p was up-regulated in OA tissues and cells. A previous study showed that miR-34a-5p was a target of SNHG7 in colorectal cancer progression [36]. In our study, we confirmed again that miR-34a-5p is a target miRNA of SNHG7 through the luciferase reporter assay. In addition, miR-34a-5p was also proved to be directly combined with SNHG7 via the RIP and pulldown assays. Interestingly, knockdown of miR-34a-5p could promote cell proliferation as well as suppress cell apoptosis and autophagy which was consistent with SNHG7 overexpression. More than that, overexpression of miR-34a-5p reversed the effect of SNHG7 on cell proliferation, apoptosis and autophagy in OA. These results showed that SNHG7 regulated OA cell growth through targeting miR-34a-5p.

Synoviolin 1 (SYVN1) was associated with ER stress, chronic inflammation, and vascular overgrowth in diabetic retinopathy (DR) [37, 38]. Overexpression of miR-125b-5p was associated with the pathogenesis of osteoarthritis by down-regulation of SYVN1 [38], reflecting that SYVN1 was closely associated with OA. Besides, in our study, SYVN1 was first identified as a target of miR-34a-5p using RIP and luciferase reporter assay. SYVN1 expression was decreased in OA tissues and cells. The expression of SYVN1 was significantly induced by anti-miR-34a-5p transfection. Functional studies showed that siSYVN1 impaired the effects of anti-miR-34a-5p on cell proliferation, apoptosis and autophagy. Meanwhile, SNHG7 transfection increased SYVN1 expression, which inhibited by miR-34a-5p overexpression, implying that SNHG7 regulated SYVN1 expression through targeting miR-34a-5p in cell progression of OA.

## Conclusion

In conclusion, we verify up-regulated SNHG7 promoted cell proliferation as well as inhibited cell apoptosis and autophagy by sponging miR-34a-5p through regulating SYVN1 for the first time, providing a novel sight to understand the pathogenesis of OA.

## Supplementary information

**Additional file 1: Figure** **S1.** SNHG7 expression was decreased in IL-1β-treated normal chondrocytes. The expression of SNHG7 in OA cells stimulated with 10 ng/ml IL-1β and OA cells. *P < 0.05.

**Additional file 2: Figure** **S2.** Overexpression of SNHG7 promoted cell proliferation and inhibited cell apoptosis in normal chondrocytes treated with IL-1β. (A) The expression of SNHG7 was detected in normal chondrocytes transfected with control, vector and lncRNA SNHG7 by qRT-PCR. (B) Cell proliferation was measured in normal chondrocytes (IL-1β) transfected with control, vector and lncRNA SNHG7 after transfection 24 h, 48 h, 72 h by MTT assay. (C) Cell apoptosis was detected in normal chondrocytes (IL-1β) transfected with control, vector and lncRNA SNHG7 by flow cytometry. *P < 0.05.

## Data Availability

All the data generated or analyzed during this study is available.

## Abbreviations

OA: Osteoarthritis
RIP: RNA immunoprecipitation
FBS: Fetal bovine serum
qRT-PCR: Quantitative real-time PCR
DR: Diabetic retinopathy
