# Supplementary figures and images for: LncRNA SNHG7/miR-34a-5p/SYVN1 axis plays a vital role in proliferation, apoptosis and autophagy in osteoarthritis
Source: Biol Res. 2020 Feb 17;53:9. doi: 10.1186/s40659-020-00275-6 (PMC7027214; doi:10.1186/s40659-020-00275-6)

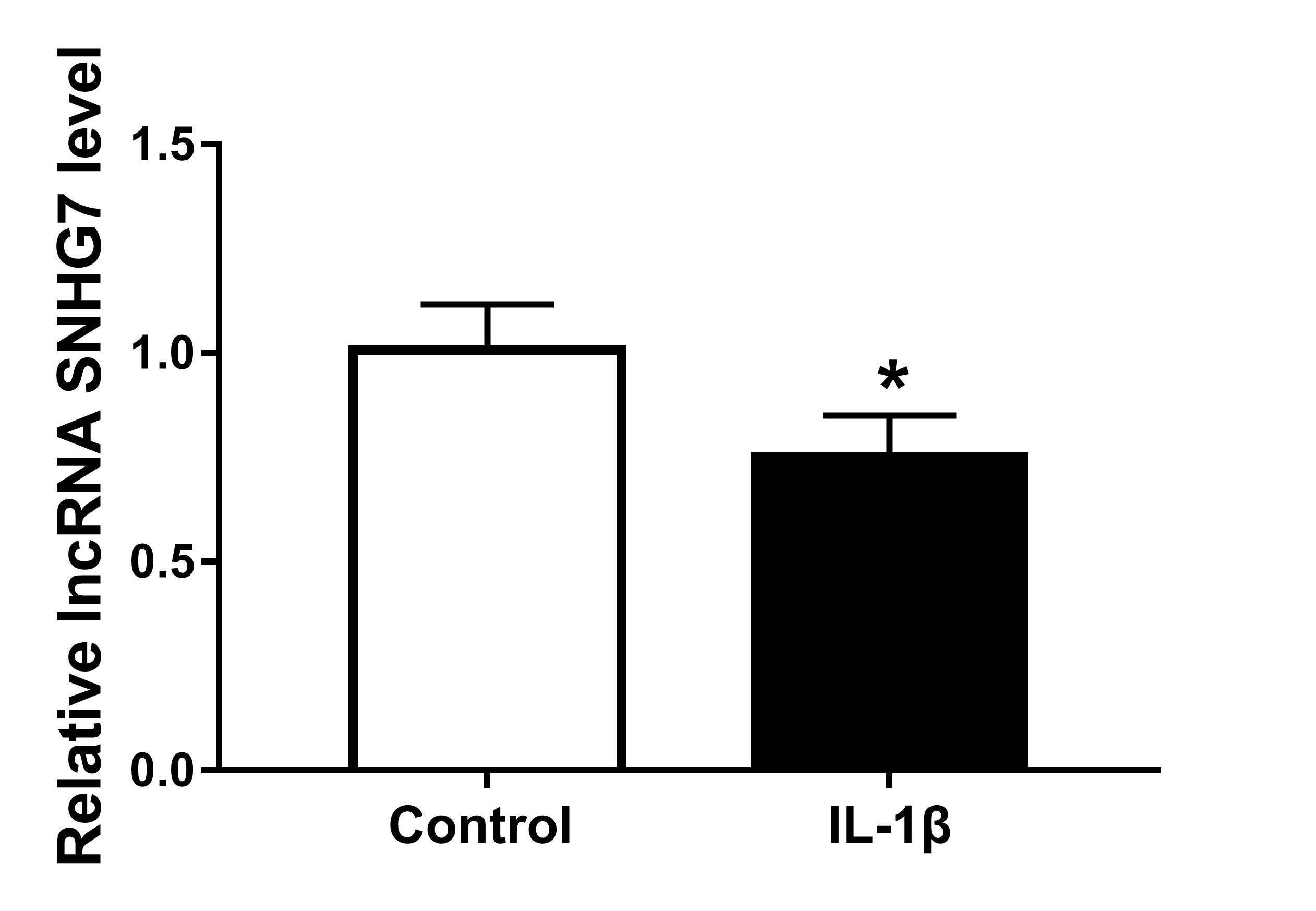

Supplement: Supplementary file 1 — Additional file 1: Figure S1. SNHG7 expression was decreased in IL-1β-treated normal chondrocytes. The expression of SNHG7 in OA cells stimulated with 10 ng/ml IL-1β and OA cells. *P < 0.05. [file 40659_2020_275_MOESM1_ESM.jpg]

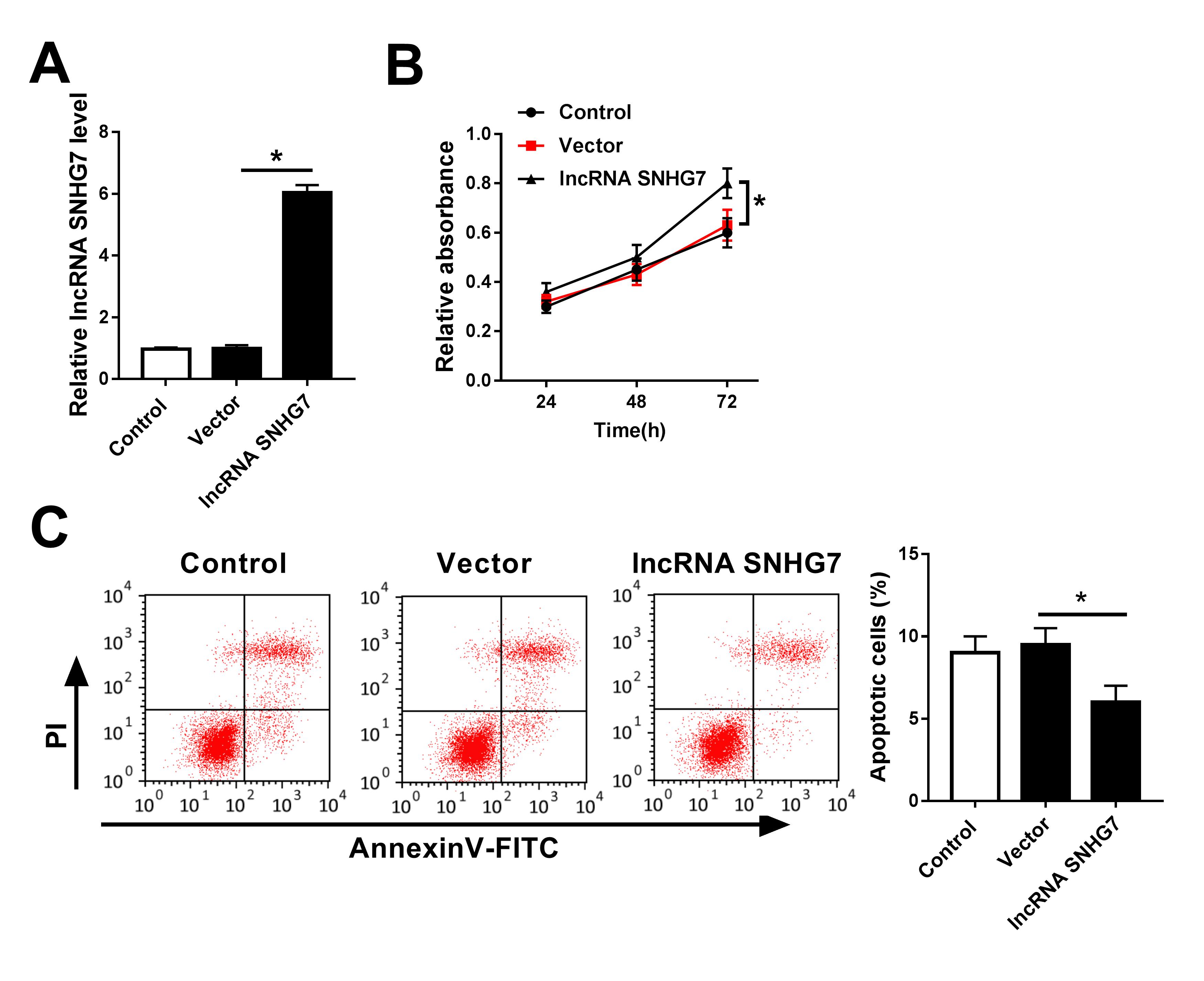

Supplement: Supplementary file 2 — Additional file 2: Figure S2. Overexpression of SNHG7 promoted cell proliferation and inhibited cell apoptosis in normal chondrocytes treated with IL-1β. (A) The expression of SNHG7 was detected in normal chondrocytes transfected with control, vector and lncRNA SNHG7 by qRT-PCR. (B) Cell proliferation was measured in normal chondrocytes (IL-1β) transfected with control, vector and lncRNA SNHG7 after transfection 24 h, 48 h, 72 h by MTT assay. (C) Cell apoptosis was detected in normal chondrocytes (IL-1β) transfected with control, vector and lncRNA SNHG7 by flow cytometry. *P < 0.05. [file 40659_2020_275_MOESM2_ESM.jpg]
